# Preoperative Lugol's solution and surgical outcomes in Graves’ disease: a single-center retrospective study

**DOI:** 10.3389/fsurg.2026.1755477

**Published:** 2026-02-26

**Authors:** Mehmet Taner Ünlü, Ozan Caliskan, Işık Çetinoğlu, Zerin Şengül, Hazal Arikan, Düşsel Gerçelman, Nurcihan Aygun, Mehmet Uludag

**Affiliations:** General Surgery Clinic, Endocrine Surgery Department, Health Science Universty Istanbul Sisli Hamidiye Etfal Training and Research Hospital, Sarıyer, Türkiye

**Keywords:** endemic goiter, Graves' disease, Lugol's solution, preoperative preparation, surgical outcomes, thyroidectomy

## Abstract

**Introduction:**

Basedow Graves disease (BG) is the most common cause of hyperthyroidism. Lugol's solution (LS) is used preoperatively to inhibit thyroid hormone production, decrease thyroid gland vascularity and ensure a safer surgical field. This study aims to evaluate efficacy of the LS and its association with surgical complications in patients with BG.

**Materials and methods:**

Patients with total thyroidectomy for BG between 2019 and 2024 were retrospectively included. Preoperative calcium (Ca), alkaline phosphatase (ALP), parathyroid hormone (PTH) levels, operative time, resected thyroid specimen weights, postoperative complications were analyzed.

**Results:**

Among 128 patients, 38(29.6%) received LS (Group1), while 90 (70.3%) did not (Group2). No significant difference was found between groups regarding preoperative Ca, ALP, PTH (*p* = 0.780, *p* = 1.000, *p* = 1.000, respectively). Mean operative times were 147.79 ± 64.66 min in Group1, 146.19 ± 35.69 min in Group2 (*p* = 0.225). Mean thyroid specimen weights were 36.83 ± 26.47 g in Group1, 43.16 ± 30.81 g in Group2 (*p* = 0.246). The rates of incidental parathyroidectomy were 21.1% (*n* = 8) in Group1 and 15.6% (*n* = 14) in Group2 (*p* = 0.456). Transient hypoparathyroidism rate was slightly higher in Group1 (34.2%, *n* = 13) than in Group2 (23.3%, *n* = 21) (*p* = 0.292). Permanent hypoparathyroidism occurred in 2.63% of Group1 and 8.89% of Group2 patients (*p* = 0.375). The rates of postoperative vocal cord paralysis, adjusted for the number of nerves at risk, were comparable between the groups (9.21%, 8.89%; *p* = 1.000).

**Conclusion:**

The primary endpoint of postoperative hypocalcemia/hypoparathyroidism did not differ significantly between groups. Although the incidence of transient hypoparathyroidism was slightly higher in the LS group, no statistically significant difference was observed. Additionally, no significant difference was found regarding intraoperative or long-term outcomes. Present study did not demonstrate a protective effect of preoperative LS on surgical complications in patients with BG.

## Introduction

Basedow-Graves disease (BG) is the most common cause of hyperthyroidism (1). It is more prevalent particularly among younger individuals and in the female population. The main treatment modalities for this disease include antithyroid drugs, radioactive iodine therapy, and surgery. Although medical therapy is most frequently preferred as the first-line treatment today, thyroidectomy with low recurrence rates may be performed in refractory BG, in patients planning pregnancy, or in those who prefer surgery over medical management (2). Despite the rapid postoperative resolution of hyperthyroidism and the low recurrence rates, the surgical approach remains a subject of debate, since complication rates are generally low but, in rare instances, have been reported to reach as high as 30%–40% in the literature (1).

Understanding the factors that complicate surgery in BG has led to the development of various preparatory treatment strategies aimed at overcoming these challenges. The clinical condition caused by abnormally elevated thyroid hormone levels, also observed in patients with BG, is termed thyrotoxicosis (3). This condition can affect multiple organ systems, most notably the cardiovascular and neuromuscular systems, and may even result in mortality (4). In addition to its systemic effects, the increased thyroid hormone secretion characteristic of BG can also lead to enhanced vascularization of the thyroid gland via neoangiogenesis, resulting in abnormally increased thyroidal blood flow. Consistent with these findings, the literature reports that thyroid blood flow in patients with BG can be increased by a factor of 3–30 compared to normal thyroid glands, and that the density of intrathyroidal microvascular structures is elevated, as confirmed by Doppler ultrasonography (5–7).

This condition observed in BG is considered one of the factors, and perhaps the most critical, that complicates surgery. Increased vascularity hinders surgical exploration and consequently increases the incidence of common thyroid surgery complications such as postoperative cervical hematoma, vocal cord paralysis, and postoperative iatrogenic hypoparathyroidism. The association between augmented thyroidal blood flow and higher complication rates remains a subject of ongoing debate in the current literature (8, 9).

Surgeons who support the view that hypervascularization increases the risk of complications argue that in conditions such as BG, which is a cause of hyperthyroidism, appropriate preoperative surgical preparation can reduce perioperative and postoperative complication rates. According to the guidelines of the American Thyroid Association (ATA), in patients with BG scheduled for thyroidectomy, euthyroidism should be achieved with methimazole whenever possible, and potassium iodide should be administered in the preoperative period. In cases requiring urgent surgery or in patients with an allergy to antithyroid drugs, preoperative preparation with beta-blockade and potassium iodide is recommended (3).

Lugol's solution (LS) is an aqueous preparation of iodine and potassium iodide, first described by Jean Lugol in 1829 (10). In patients undergoing thyroidectomy for BG, LS is typically administered for a short period prior to surgery. The primary aim of this preoperative administration is to reduce the vascularity of the thyroid gland, thereby limiting intraoperative bleeding and preventing surgical complications (11). The literature has demonstrated that LS temporarily inhibits thyroid hormone synthesis, decreases hormone release, and blocks the organification of iodine (12–16). Moreover, LS has also been shown to reduce angiogenesis and inflammation markers such as vascular endothelial growth factor (VEGF) and interleukin-16 (IL-16) (17–21).

However, the effect of LS on surgical outcomes remains controversial; while some studies have reported no significant reduction in intraoperative bleeding and morbidity, others have demonstrated a marked decrease (1, 8, 22).

Although current evidence demonstrates that LS confers significant biochemical and hemodynamic benefits, further research is required to more clearly elucidate its effects on surgical outcomes.

Many guidelines, including those of the ATA, as well as numerous endocrine surgeons, recommend the use of LS in patients with BG; however, the absence of a clear consensus in the literature has left this issue unresolved. In light of this gap, the present study aimed to retrospectively evaluate the effectiveness of LS in the surgical management of BG and its association with surgical complications.

## Material - method

In this retrospective study, 128 patients diagnosed with BG who underwent total thyroidectomy in the Department of General Surgery at our hospital between 2019 and 2024 were evaluated. Inclusion criteria were a confirmed diagnosis of BG (based on imaging and biochemical assessments), performance of total thyroidectomy or the Dunhill procedure, and complete availability of patient medical records. Patients were excluded if they had undergone lobectomy, hemithyroidectomy, or subtotal thyroidectomy; had surgery for other thyroid pathologies; underwent parathyroid autotransplantation; received additional surgical procedures concomitant with BG surgery; failed to achieve preoperative euthyroidism; or had incomplete medical documentation.

All operations were performed in our hospital by four surgeons specialized in endocrine surgery, with the aid of intraoperative neuromonitoring. Details of the surgical technique have been described in previous publications. All surgeries were performed with intraoperative neuromonitoring, utilizing either the intermittent or the continuous modality. In cases where the parathyroid glands could not be visualized, no additional dissection was performed (23–26).

Patients were divided into two groups based on whether LS was administered preoperatively. Group 1 consisted of 38 patients who received LS starting seven days prior to surgery, while Group 2 included 90 patients who did not receive LS. In all patients, preoperative serum levels of triiodothyronine (T3), thyroxine (T4), thyroid-stimulating hormone (TSH), calcium (Ca), alkaline phosphatase (ALP), and parathyroid hormone (PTH) were recorded. Postoperative measurements included Ca, ALP, and PTH levels at the 2nd hour and on postoperative day 1, as well as operative time (minutes), weight of the excised thyroid tissue (grams), and the presence of inadvertently excised parathyroid glands in pathology specimens, as documented in medical records and pathology reports.

In our study, all patients in the LS group received oral LS (classic formulation containing 5% iodine and 10% potassium iodide) according to a prespecified, standardized preoperative protocol, consistent with guideline based practice (3). LS was administered as a fixed dose of 5 drops orally three times daily (≈120 mg iodide/day, based on the ATA estimate of 8 mg iodide per drop), initiated 7 days before surgery and continued without interruption until the day of operation, within the range of commonly used regimens reported in the literature and with a similar administration schedule to a recent randomized clinical trial (1, 3). The initiation date, dosing schedule, and treatment duration were verified from medication administration records and clinical charts. Eligibility for the LS group required documented completion of the full 7 day standardized regimen with uninterrupted administration up to surgery. The decision to initiate preoperative LS therapy was made at the discretion of the attending surgeon, based on individual clinical judgment and daily practice patterns, rather than predetermined standardized clinical or biochemical criteria. Consequently, patient grouping reflected the natural variability of routine clinical practice rather than strict protocol-driven selection. Since the decision to administer LS was made according to the attending surgeon's clinical judgment, some variability in patient characteristics between groups may have been introduced. This is recognized as a limitation of our work. The initiation date, dosing schedule, and treatment duration were verified from patient medical records, and inclusion in the LS group required initiation of treatment at least seven days before surgery and uninterrupted continuation until the day of operation. The non-LS group (Group 2) comprised patients undergoing total thyroidectomy for BG during the same period who had not received any form of preoperative iodine supplementation.

Only patients who achieved euthyroidism in the preoperative period were included in our study. Patients with subclinical hyperthyroidism, overt hyperthyroidism, subclinical hypothyroidism, or overt hypothyroidism were excluded. In all patients, serum T3, T4, and TSH levels were measured within 72 h prior to surgery, and those whose values were within normal reference ranges were considered euthyroid.

In the postoperative period, serum Ca and PTH levels were measured within the first 6 h, within the first 24 h, and at the 6th postoperative month, and the biochemical responses of the two groups were compared. A PTH level below the reference range within the first 72 h postoperatively was considered acute hypoparathyroidism. Postoperative hypoparathyroidism was further classified as transient or permanent; transient hypoparathyroidism was defined as postoperative hypocalcemia resolving within 6 months, whereas permanent hypoparathyroidism was defined as hypocalcemia persisting beyond this period (27).

Vocal cord function was assessed in the postoperative period by an otorhinolaryngologist using indirect laryngoscopy, and the presence and laterality of vocal cord paralysis were recorded. Pathology reports were reviewed to determine whether inadvertently excised parathyroid tissue was present within the resected thyroid specimens.

All data were obtained retrospectively from patient medical records. The study was conducted in accordance with the Declaration of Helsinki and was approved by the local ethics committee. Although the study was retrospective in design, written informed consent for the use of clinical data in research and for potential publication had been routinely obtained from all patients as part of institutional policy.

Primary and secondary outcomes: The primary endpoint of the study was the incidence of postoperative hypocalcemia/hypoparathyroidism (transient and permanent) after total thyroidectomy for BG. Secondary endpoints included operative time, rates of vocal cord paralysis, incidence of incidental parathyroidectomy, and longitudinal changes in serum Ca, PTH, and ALP levels in the early and late postoperative period.

Statistical Analysis: Statistical Analysis: Descriptive statistics of the data were presented using mean, standard deviation, median, minimum, maximum, frequency, and percentage values. The distribution of variables was assessed using the Kolmogorov–Smirnov and Shapiro–Wilk tests. For the analysis of normally distributed quantitative independent variables, the Independent Samples *t*-test was used, while the Mann–Whitney *U*-test was applied for non-normally distributed quantitative independent variables. Categorical independent variables were analyzed using the Chi-square test, and when the assumptions of the Chi-square test were not met, the Fisher's exact test was used. All statistical analyses were performed using SPSS 28.0 software.

## Results

Of the 128 patients who met the inclusion criteria, 101 (78.9%) were female and 27 (21.1%) were male (Table 1). In 38 patients (29.6%) (Group 1), LS was administered, whereas 90 patients (70.3%) (Group 2) did not receive LS (Table 2). Group 1 consisted of 38 patients, including 35 females and 3 males, while Group 2 included 90 patients, 66 of whom were female and 24 were male (Table 2). The mean age of the study population was 43.1 ± 13.0 years (range, 15–79 years); the mean age was 40.9 ± 12.2 years (range, 17–75 years) in Group 1 and 44.1 ± 13.3 years (range, 15–79 years) in Group 2 (Table 2). No statistically significant difference was observed in age distribution between the groups (*p* = 0.204) (Table 2). Similarly, no significant differences were found between the two groups in preoperative serum Ca, ALP, and PTH levels (*p* = 0.780, *p* = 0.809, and *p* = 1.000, respectively) (Table 3). Preoperative indirect laryngoscopic examination revealed normal bilateral vocal cord mobility in all patients.

**Table 1:** Demographic findings of all patients.

Age		Min-Max	Median	Mean ± SD
		15.0–79.0	42.9	43.1 ± 13.0
Gender	Male	27 (21.1%)		128
	Female	101 (78.9%)		

**Table 2:** Number of patients, their genders, and mean ages by groups.

Characteristic		Group 1 (With LS)	Group 2 (Without LS)	*p*-value
Number of patients		38 (29.6%)	90 (70.3%)	N/A
Gender	M	3 (7.9%)	66 (73.3%)	N/A
	F	35 (92.1%)	24 (26.7%)	
Mean age		40.9 ± 12.2 (17–75)	44.1 ± 13.3 (15–79)	0.204

**Table 3:** Preoperative, peroperative and postoperative findings by groups.

Preoperative, peroperative and postoperative findings	Group 1 (With LS)	Group 2 (Without LS)	*p*-value
Duration of surgery (min)	147.8 ± 64.7	146.2 ± 35.7	0.225
Patients in whom the parathyroid gland was incidentally identified in the pathology specimen	8 (%21.1)	14 (%15.6)	0.456
Preoperative calcium (mg/dl)	9.42 ± 0.51	9.51 ± 0.48	0.7801
Preoperative PTH (pg/ml)	54.38 ± 27.37	54.97 ± 25.73	1.0000
Preoperative ALP (U/L)	106.28 ± 71.14	95.92 ± 51.49	0.8091
Postoperative 2nd hour Calcium (mg/dl)	8.57 ± 0.53	8.81 ± 0.52	0.0220
Postoperative 2nd hour ALP (U/L)	93.36 ± 65.15	85.24 ± 43.92	0.9955
Postoperative 2nd hour PTH (pg/ml)	25.60 ± 21.36	25.55 ± 21.27	0.8520
Postoperative day 1 Calcium (mg/dl)	9.42 ± 0.51	9.51 ± 0.48	0.283
Postoperative day 1 PTH (pg/ml)	35.14 ± 73.81	27.82 ± 18.71	0.2583
Postoperative 6th month PTH (pg/ml)	46.14 ± 31.25	43.73 ± 23.59	0.375
Transient hypoparathyroidism (%)	13 (34.2%)	21 (23.3%)	0.292
Permanent hypoparathyroidism (%)	1 (2.63%)	8 (8.89%)	0.375
Vocal cord paralysis (by the number of nerves at risk) (%)	7/76 (9.21%)	16/180 (8.89%)	1.000

The mean operative times (from skin incision to wound closure) in Group 1 and Group 2 were 147.79 ± 64.66 min and 146.19 ± 35.69 min, respectively (*p* = 0.225). The mean weights of the excised thyroid specimens were 36.83 ± 26.47 g in Group 1 and 43.16 ± 30.81 g in Group 2 (*p* = 0.246). The incidence of inadvertently excised parathyroid glands identified in the pathology specimens was 21.1% (*n* = 8) in Group 1 and 15.6% (*n* = 14) in Group 2, with no statistically significant difference between the groups (*p* = 0.456) (Table 3).

In the early postoperative period (2 h after surgery), a statistically significant difference was observed between the groups in terms of serum Ca levels (*p* = 0.022) (Table 3). The mean serum Ca level at this time point was 8.57 ± 0.53 mg/dl in Group 1 and 8.81 ± 0.52 mg/dl in Group 2. In contrast, no significant differences were found in the concomitantly measured PTH and ALP values (*p* = 0.852 and *p* = 0.995, respectively) (Table 3). Although this difference reached statistical significance, both values were within the normal reference range and were not clinically meaningful. In our institution, measurement of serum calcium at the 2nd postoperative hour is performed routinely for other clinical and research purposes; however, its value in predicting postoperative hypocalcemia is limited. For the present study, the finding was reported because it showed statistical significance, although it did not influence clinical management or outcomes.

On postoperative day 1, mean serum Ca levels were 9.42 ± 0.51 mg/dl in Group 1 and 9.51 ± 0.48 mg/dl in Group 2 (*p* = 0.283), while mean PTH levels were 34.07 ± 68.58 pg/ml and 26.35 ± 18.94 pg/ml, respectively (*p* = 0.499) (Table 3).

At postoperative month 6, mean serum Ca levels were 9.18 ± 0.75 mg/dl in Group 1 and 9.23 ± 0.66 mg/dl in Group 2, with no statistically significant difference between the groups (*p* = 0.930) (Table 3). Mean PTH levels at the same time point were 46.14 ± 31.25 pg/ml in Group 1 and 43.73 ± 23.59 pg/ml in Group 2, also without a significant difference (*p* = 0.375) (Table 3). The incidence of transient hypoparathyroidism was 34.2% (*n* = 13) in Group 1 and 23.3% (*n* = 21) in Group 2, with no significant intergroup difference (*p* = 0.292) (Table 3). Permanent hypoparathyroidism occurred in 2.63% of patients in Group 1 and 8.89% in Group 2, again without a statistically significant difference (*p* = 0.375) (Table 3). When analyzed according to the number of nerves at risk, the rates of postoperative vocal cord paralysis were comparable between Group 1 and Group 2 (9.21% vs. 8.89%, *p* = 1.000) (Table 3).

## Discussion

The use of LS in thyrotoxic patients is preferred in situations where antithyroid drugs are insufficient to achieve adequate control, as it enables rapid suppression of thyroid hormone release. The effects of LS on the thyroid can be explained by two primary mechanisms: the Wolff–Chaikoff effect and the Plummer effect. The Wolff–Chaikoff effect is an autoregulatory response in which high iodine intake transiently inhibits thyroid hormone synthesis. In this mechanism, the organification step of iodine within the thyroid is blocked, leading to a temporary cessation of T3 and T4 production. In normal thyroid tissue, this inhibitory effect resolves within a few days through the development of the so-called “escape phenomenon”, allowing hormone synthesis to resume. The Plummer effect, on the other hand, is most notably observed in BG and is characterized by the rapid suppression of thyroid hormone release in addition to the inhibition of hormone synthesis. This effect forms the primary therapeutic rationale for the preoperative use of LS, as it allows for a clinically significant reduction in hormone levels within a few days in thyrotoxic patients. In this context, the Wolff–Chaikoff effect represents a physiological protective reflex, whereas the Plummer effect serves as the basis for an active therapeutic intervention. The preoperative use of LS is primarily aimed at exploiting these mechanisms. Although our study does not provide direct data on preoperative FT3 and FT4 levels, these mechanisms have been described in detail at the biochemical level in the literature and constitute a major rationale for the preoperative use of LS (13, 28, 29).

Beyond being one of the most important tools for achieving euthyroidism in the preoperative period for patients with BG, LS has also been investigated in numerous studies for its potential intraoperative benefits. Current guidelines emphasize the need for more experienced surgeons in BG surgery, noting that it remains a more challenging surgical procedure compared to most other benign thyroid diseases (3). In particular, the abnormal increase and fragility of vascularization in BG lead to greater thyroid gland perfusion, which can cause significant intraoperative bleeding and make exploration and dissection more difficult. These challenges, even in the hands of highly experienced endocrine surgeons, can translate into higher perioperative and postoperative complication rates (3). To ensure both morphological and functional preservation of the recurrent laryngeal nerve, intraoperative neuromonitoring techniques are widely employed; similarly, to reduce postoperative hypoparathyroidism, adjunctive intraoperative imaging methods are utilized, and surgical loupes are frequently adopted to allow more precise dissection (30–32). While these adjunctive techniques can be applied to all thyroid surgeries, among preoperative strategies specifically aimed at reducing surgical complications in BG patients, LS administration remains one of the most widely adopted and popular methods.

In our study, no statistically significant difference in operative time was observed between patients who received LS and those who did not. The mean surgical duration was 147.8 ± 64.7 min in Group 1 and 146.2 ± 35.7 min in Group 2 (*p* = 0.225). This finding suggests that, despite the theoretical expectation that LS may facilitate surgical dissection by reducing thyroid vascularity, its practical impact on operative time appears negligible. The literature reports conflicting results on this matter. For instance, in a randomized controlled trial by Whalen et al., the use of saturated solution of potassium iodide (SSKI) in BG patients was associated with a significantly shorter operative time (33). Conversely, Tsai et al., in a systematic review, reported that while LS may modestly reduce intraoperative blood loss, it has no significant effect on operative duration (34). The results of our study are consistent with these heterogeneous findings, indicating that the potential technical advantages of LS do not translate into a measurable reduction in operative time. This raises questions about the necessity of routine preoperative LS administration and suggests that its use may be better reserved for individualized, patient-specific strategies.

Hypoparathyroidism is among the most common complications of thyroid surgery and represents a particularly significant risk following total thyroidectomy for BG. In this patient group, inadvertent resection of the parathyroid glands or disruption of their vascular supply can result in functional loss and permanent hypocalcemia (35). Increased glandular vascularization and the technical difficulty of surgical dissection in BG further elevate the risk of parathyroid injury (36–38). One of the leading causes of postoperative hypoparathyroidism is the unintentional removal of parathyroid glands during thyroidectomy. In BG, the increased vascularity and the greater difficulty of exploration and dissection may be expected to increase the number of inadvertently excised parathyroid glands, consequently raising the risk of postoperative hypoparathyroidism and hypocalcemia.

In our study, administration of LS had no significant impact on rates of postoperative hypoparathyroidism (temporary or permanent), hypocalcemia, or the number of inadvertently removed parathyroid glands. The most notable finding was the statistically significant difference in early postoperative serum Ca levels between the groups (*p* = 0.0220). At postoperative hour 2, Ca levels were significantly lower in Group 1 compared with Group 2 (8.57 ± 0.53 mg/dl vs. 8.81 ± 0.52 mg/dl). Although the absolute difference was relatively small, this may hold clinical relevance for early postoperative patient management. At postoperative day 1 and month 6, Ca and PTH values showed no statistically significant differences, though results in Group 1 tended to be slightly lower.

Contrary to the common expectation among surgeons, our findings align with those of Hedberg et al., who reported similar outcomes in a large retrospective cohort of 813 BG patients (22). They suggested that patients receiving LS might have more severe disease, a shorter duration in the euthyroid state, and technically more challenging dissections, potentially explaining this phenomenon. However, in both their study and ours, operative times were similar between LS and non-LS groups, weakening the argument that more difficult dissection accounts for the results. Likewise, in our study—as in Hedberg et al.—thyroid specimen weights were comparable between groups, further suggesting that dissection difficulty was not substantially different.

Ali et al., in a retrospective study of 266 BG patients undergoing total thyroidectomy, similarly found no significant effect of LS on rates of temporary or permanent hypocalcemia/hypoparathyroidism (39). Our findings therefore support heightened vigilance for hypocalcemia in LS-treated patients during the postoperative period. In clinical practice, preoperative LS administration could be accompanied by prophylactic Ca and/or vitamin D analog supplementation to mitigate this risk.

One of the most serious complications of thyroid surgery is recurrent laryngeal nerve (RLN) injury, which can result in vocal cord paralysis (VCP) and significantly influence postoperative morbidity. This complication may lead to symptoms such as hoarseness, dysphagia, aspiration, and, in rare cases of bilateral paralysis, respiratory failure. The literature highlights that the incidence of RLN injury can be higher in thyroidectomies performed for BG (40, 41).

In our study, the rates of vocal cord paralysis (VCP) in patients who received and did not receive preoperative LS were 9.21% and 8.89%, respectively, with no statistically significant difference between the groups (*p* = 1.000). When evaluated according to the number of nerves at risk, no difference was observed. This suggests that, despite the theoretical advantage of LS in reducing thyroid gland vascularity and facilitating dissection, it does not significantly decrease the risk of RLN injury. The overall incidence of VCP in our cohort was approximately 9%, which lies at the upper limit of the rates reported in the literature (3%–8%). This can be attributed to the fact that our series exclusively included patients with BG, a condition that makes thyroidectomy technically more challenging due to increased vascularity, glandular fragility, and perithyroidal changes. Therefore, the slightly higher rate of VCP in our study likely reflects the inherent surgical difficulty of this specific patient population rather than differences in surgical quality (40, 42).

Several BG related factors may sustain RLN injury risk even with LS: pronounced hyperplasia and hypervascularity, intraoperative bleeding that limits visibility, gland fragility increasing nerve contact during manipulation, occasional RLN anatomical variation, and perithyroidal inflammation or fibrosis impairing nerve identification. Therefore, reduced vascularity may not yield a meaningful reduction in RLN injury rates.

Our findings are consistent with those of Hedberg et al., who reported no significant difference in RLN injury with vs. without LS (22), and with the systematic review by Tsai et al., which likewise found no protective effect (34). All procedures in our study were performed with intraoperative neuromonitoring by experienced endocrine surgeon, which may partly explain the low complication rates; moreover, LS may have limited clinical impact if its vascularity reducing effect is mainly microvascular and/or if glandular edema persists, especially in severe BG. In conclusion, our study found that the administration of LS had no significant effect on the incidence of vocal cord paralysis. This finding maintains the ongoing debate regarding the protective role of LS against RLN injuries and supports the view that the primary determinants in preventing this complication are the surgeon's experience, surgical technique, and the use of adjunctive technologies such as intraoperative neuromonitoring. In our study, 76 nerves at risk were examined in Group 1 and 180 in Group 2, suggesting that studies with a greater number of nerves at risk may provide a clearer assessment of the impact of LS on vocal cord paralysis.

The main limitations of our study include its retrospective design, single-center setting, and relatively small sample size. Consequently, a formal power analysis could not be performed, and the results should be interpreted as preliminary findings from an observational cohort. However, the total number of BG patients included (*n* = 128) is comparable to, or larger than, many previously published single-center series evaluating preoperative LS in Graves' disease, suggesting that the study is reasonably powered to detect clinically meaningful differences in the primary endpoint at the cohort level. Moreover, the limited sample size precluded the use of multivariable regression analyses to adjust for potential confounders such as age, sex, gland size, disease severity, and surgeon, which represents another limitation. Although no statistically significant differences were observed, the lack of multivariable analysis limits our ability to control for potential confounding. Therefore, subtle but clinically meaningful effects of LS administration cannot be fully excluded. In addition, the effect of LS on thyroidal vascularity was assessed indirectly through perioperative and postoperative parameters rather than by objective imaging modalities such as Doppler ultrasonography, a constraint inherent to the retrospective nature of the study. Similarly, intraoperative variables such as blood loss, vascularity scoring and transfusion requirements were not consistently available in the medical records or systematically recorded in the surgical notes, limiting our ability to directly evaluate LS's effect on intraoperative hemostasis. These aspects could be more comprehensively evaluated in a prospective study with a larger sample size and standardized data collection. Given the retrospective nature of the study, LS administration was based on surgeon preference rather than predefined criteria, reflecting real world clinical practice. While this non randomized study provides valuable insights into routine care patterns, it inherently carries a potential for selection bias, which should be acknowledged when interpreting group comparisons. In this respect, the present work may be regarded as a preliminary contribution to the field.

In this context, the consistent finding of “no significant difference” between groups should be interpreted with caution, as the limited sample size may have reduced the ability to detect subtle but clinically relevant effects. Nevertheless, by focusing exclusively on BG patients and reflecting real world patterns of LS administration, our study provides preliminary but meaningful insights that may inform future larger, prospective trials.

Despite these limitations, our work has notable strengths: it represents one of the larger single-center cohorts on this topic; all patients were biochemically euthyroid preoperatively, minimizing confounding; surgical procedures were performed using a standardized technique by a dedicated endocrine surgery team; and follow-up was comprehensive with detailed complication profiling. We believe these methodological strengths enhance the reliability of our findings and ensure their relevance to current surgical practice. Given the ongoing debate and limited high-quality evidence regarding preoperative LS use in BG, our study provides meaningful insights and supports the need for larger, prospective, multicenter trials to further inform clinical guidelines.

As a conclusion; our study aimed to evaluate the impact of preoperative LS administration on surgical outcomes in patients undergoing thyroidectomy for BG. The findings demonstrated no statistically significant differences between the LS and non-LS groups in terms of operative time, postoperative hypoparathyroidism, or vocal cord paralysis. However, the relatively lower Ca levels observed in the LS group during the early postoperative period suggest a potentially increased Ca requirement in this patient cohort. This finding indicates the need for closer postoperative monitoring in patients receiving LS and suggests that prophylactic Ca and/or vitamin D supplementation may be beneficial in selected cases. Although our results show that LS does not provide an absolute benefit in reducing surgical complications, they highlight the importance of individualized surgical preparation in BG and underscore the need for prospective studies with stronger methodological designs to further clarify its role. Given the absence of measurable surgical benefit in our cohort, routine use of LS in euthyroid BG patients may need to be reconsidered in favor of individualized preoperative strategies.

## Data Availability

The raw data supporting the conclusions of this article will be made available by the authors, without undue reservation.
